# Supplementary material for: Quantifying negative selection in human 3ʹ UTRs uncovers constrained targets of RNA-binding proteins
Source: Nat Commun. 2024 Jan 2;15:85. doi: 10.1038/s41467-023-44456-9 (PMC10762232; doi:10.1038/s41467-023-44456-9)
Supplement: Supplementary file 2 — Supplementary Figures [file 41467_2023_44456_MOESM2_ESM.pdf]

Quantifying negative selection in human 3' UTRs uncovers constrained targets of RNA-binding proteins

Supplementary Figures

a

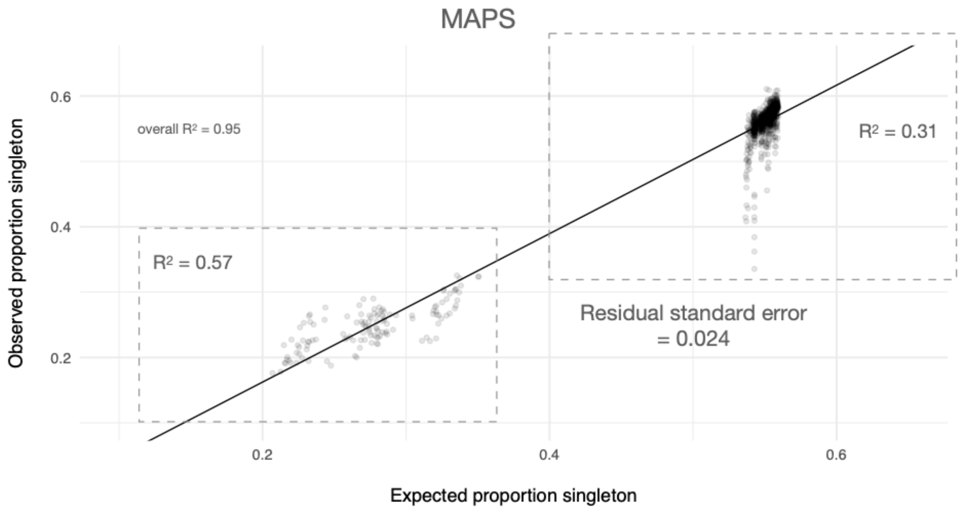

b

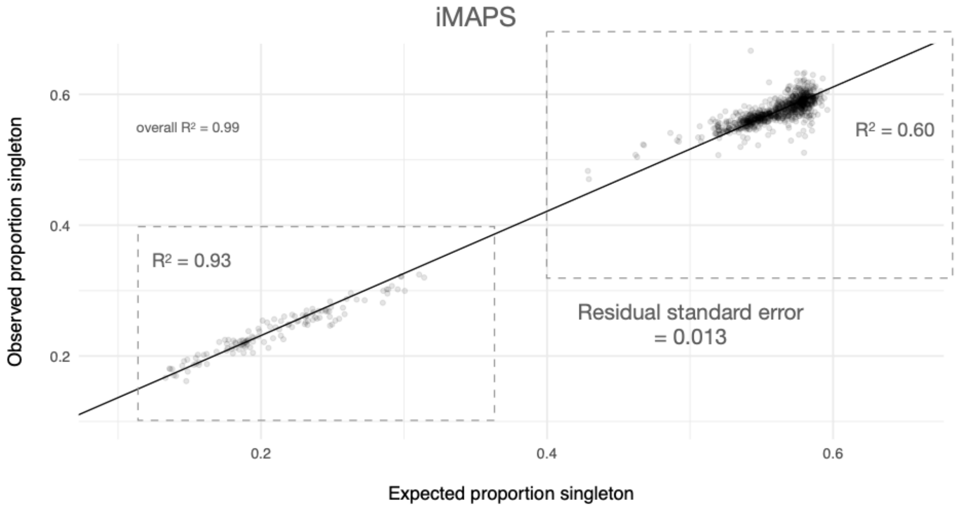

c

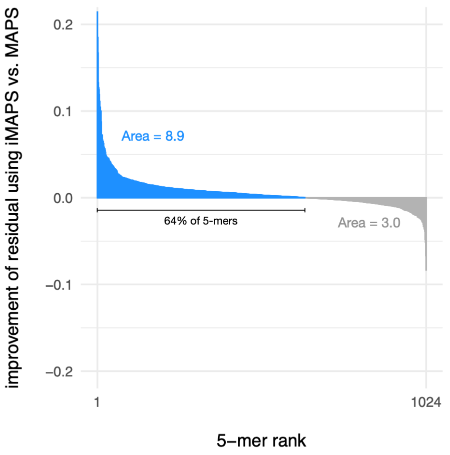

Supplementary Fig. 1: Comparing expected proportion singleton values from MAPS (Whiffin et al., 2018) and iMAPS to observed proportion singleton values for all 5-mers in 3' UTRs. Under the premise that the vast majority of occurrences of the vast majority of all possible 5-mers are not under strong negative selection in 3' UTRs, a good model of how non-selective forces influence proportion singleton values should explain much of the variance in the observed proportion singleton values across 5-mers. When we fit linear models for the MAPS approach (a) and the iMAPS approach (b), iMAPS resulted in higher correlations, an over 1.8-fold reduction in residual standard error, and the majority of 5-mers with regression residuals closer to 0 relative to MAPS (c).

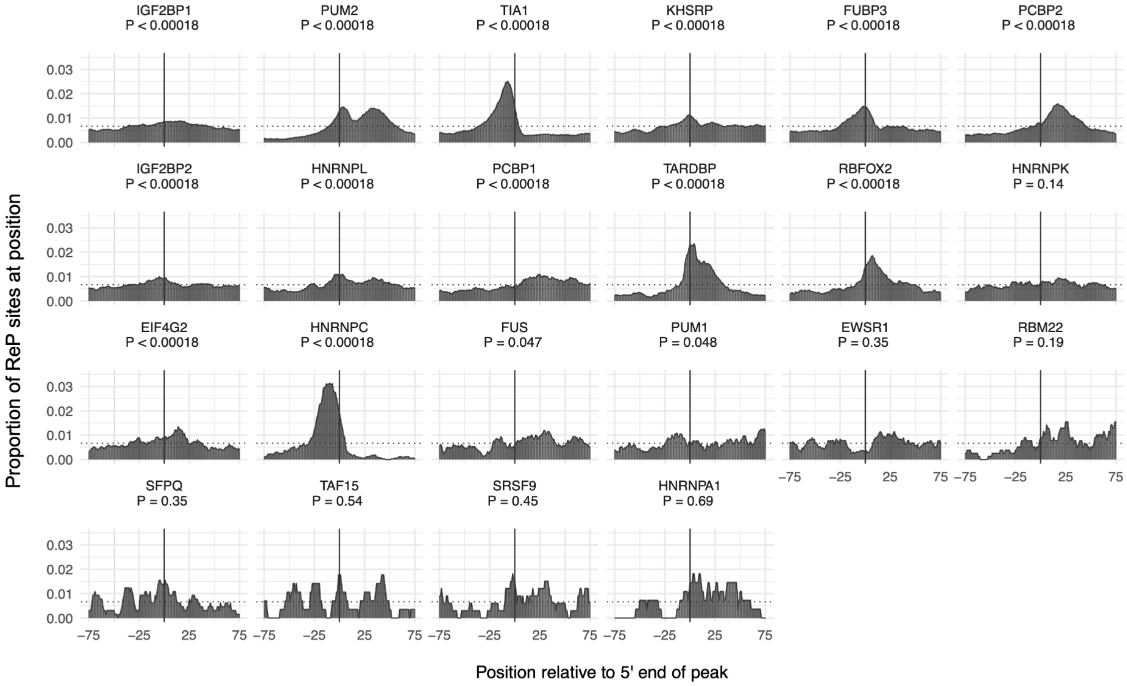

Supplementary Fig. 2: ReP sites are typically located close to the 5' end of eCLIP peaks in 3' UTRs. The union of eCLIP peaks across both HepG2 and K562 cell lines was used for RBPs with clip data in both cell lines. RBPs are sorted in order of descending number of peaks in 3' UTRs. Vertical lines indicate eCLIP peak 5' ends. The dashed horizontal lines indicate the null expectation of uniformly distributed highest affinity sites relative to eCLIP peaks. P values represent the proportion of 10,000 simulations with a maximum proportion value greater than the actual observed value, after Benjamini-Hochberg adjustment for multiple hypothesis testing. Number of peaks for each RBP: IGF2BP1, n = 5,024; PUM2, n = 4,498; TIA1, n = 3,598; KHSRP, n = 2,807; FUBP3, n = 2,413; PCBP2, n = 2,288; IGF2BP2, n = 1,642; HNRNPL, n = 1,435; PCBP1, n = 1,276; TARDBP, n = 965; RBFOX2, n = 890; HNRNPK, n = 887; EIF4G2, n = 430; HNRNPC, n = 346; FUS, n = 305; PUM1, n = 240; EWSR1, n = 186; RBM22, n = 88; SFPQ, n = 59; TAF15, n = 36; SRSF9, n = 32; HNRNPA1, n = 27; TRA2A, n = 12.

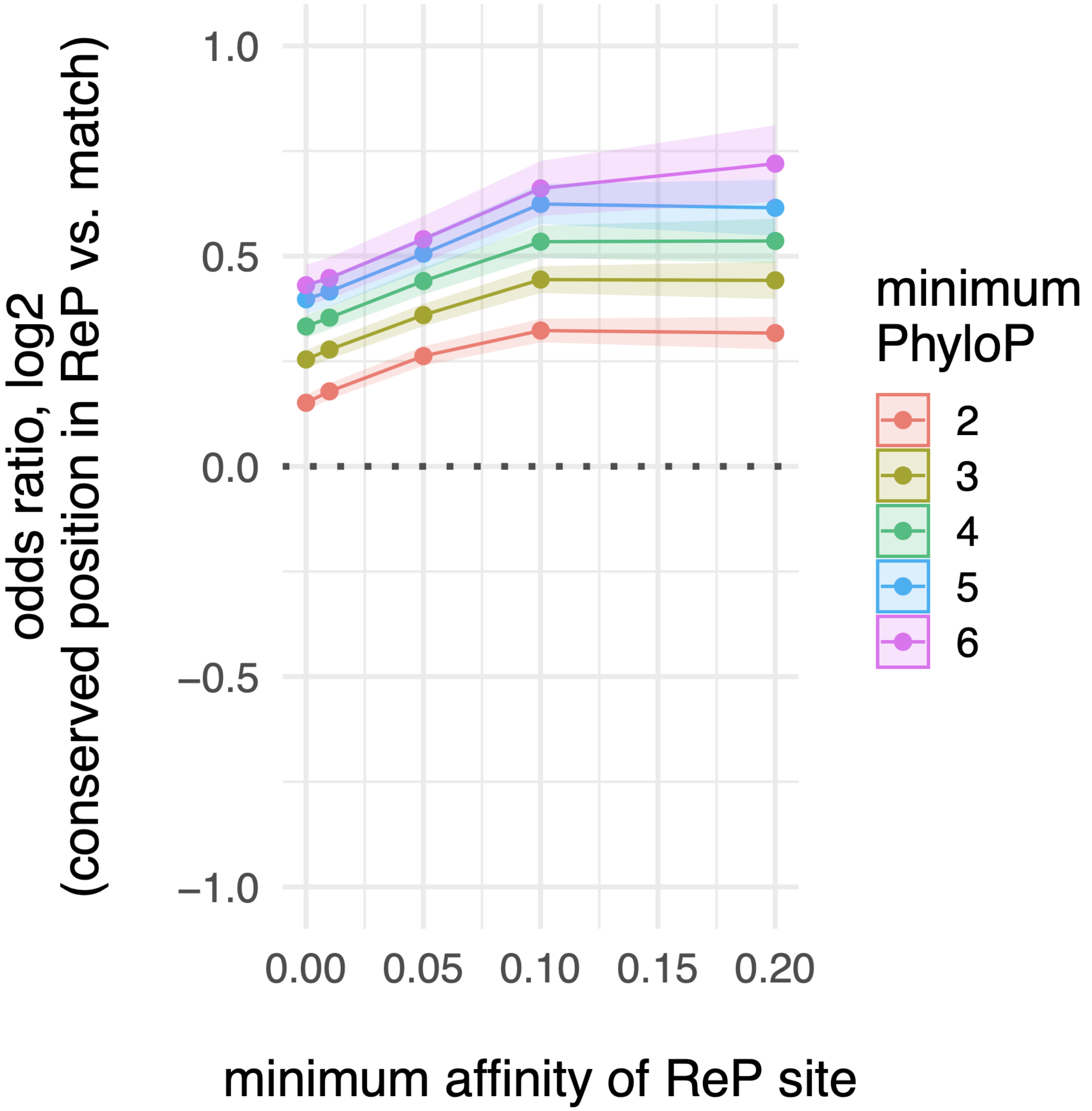

Supplementary Fig. 3: Positions within ReP sites (n = 282,685) are more likely to be conserved across species than position-matched eCLIP peak region positions (n = 282,685). Shaded regions indicate 95% confidence intervals. The horizontal dashed line marks equal likelihood of conservation.

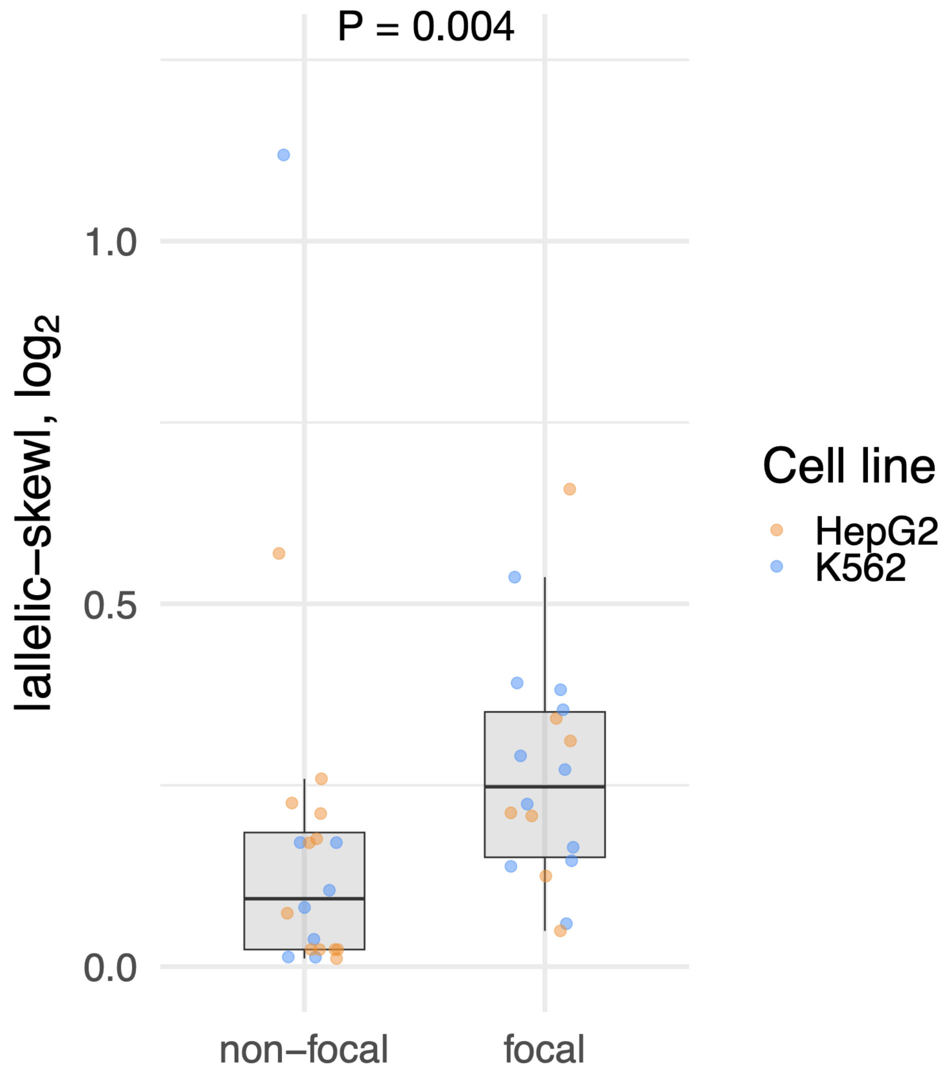

Supplementary Fig. 4: Focal ReP site variants (n = 36) promoted significantly larger skews in steady-state transcript levels than non-focal ReP site variants (n = 40) in a 3' UTR MPRA. P value is the result of a Wilcoxon rank sum test with continuity correction.

a

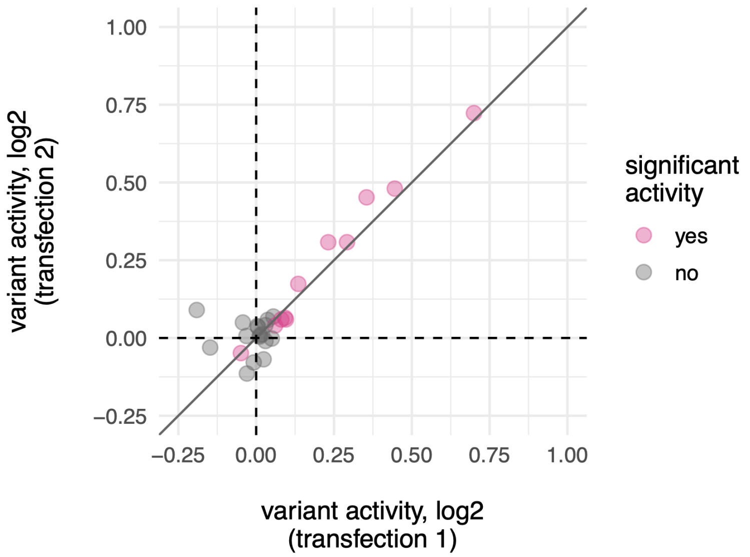

b

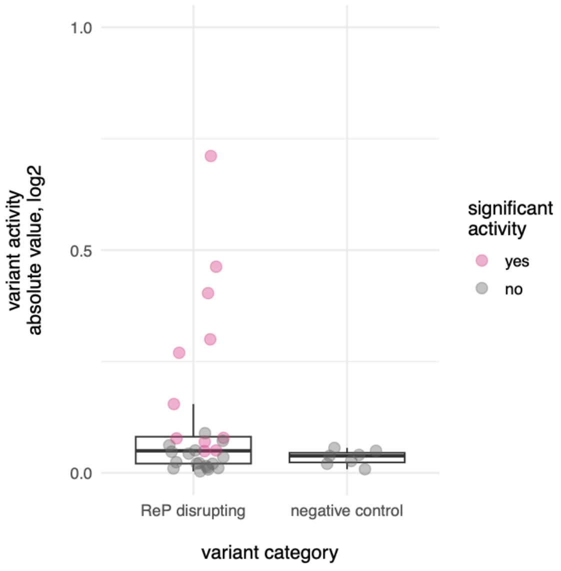

Supplementary Fig. 5: ReP site-disrupting 3' UTR variants frequently directly modulate transcript levels in cells. a) ReP site-disrupting variants (n = 28) frequently have reproducible activity across two separate transfections. The solid line highlights equal activity in each transfection. b) ReP site-disrupting variant activity is generally greater than activity of negative control variants (n = 7) not expected to modulate transcript levels (see Methods).

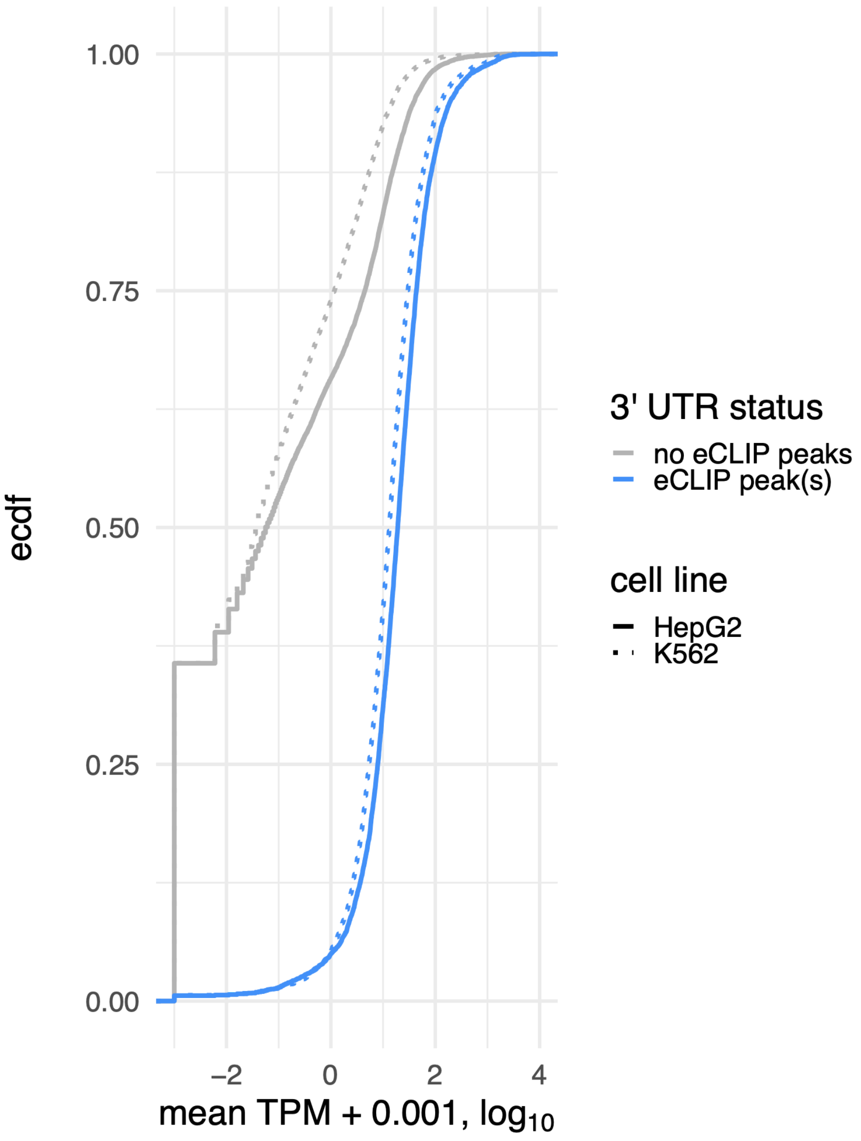

Supplementary Fig. 6: Transcripts with 3' UTR eCLIP peaks (for any RBP) are more highly expressed in HepG2 and K562 cells. TPM = transcripts per million. Values shown are means across two replicates for each cell line. Number of genes: HepG2, n = 6,805 with 3' UTR eCLIP peaks and n = 13,080 without. K562, n = 7,360 with and n = 12,525 without.

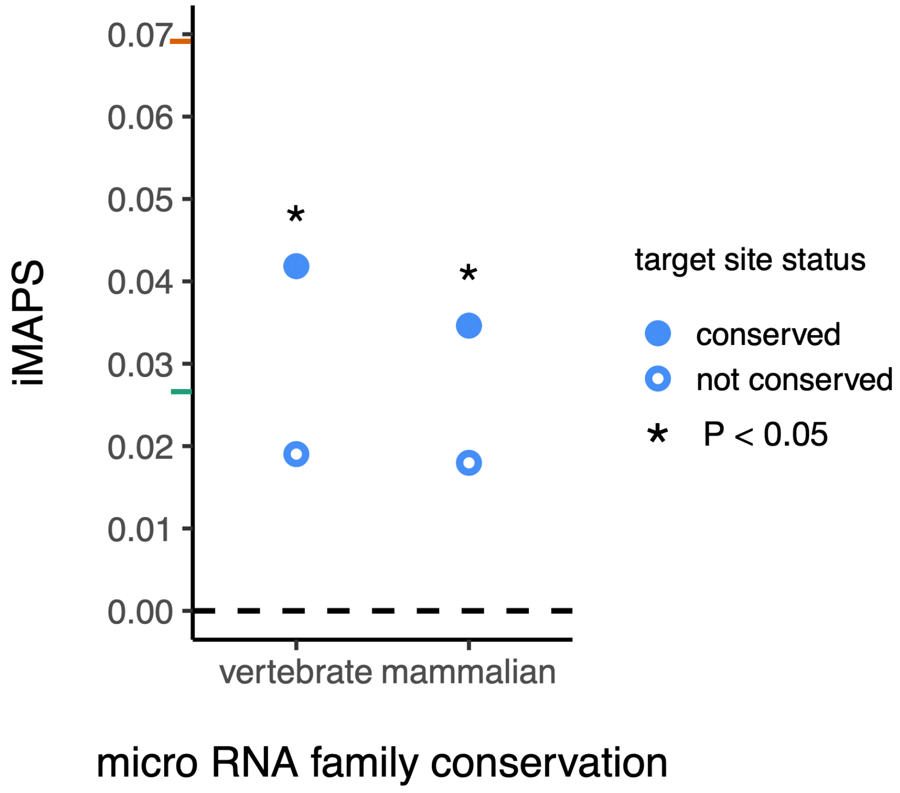

Supplementary Fig. 7: Conserved miRNA targets are under stronger selection than non-conserved targets. Asterisks indicate one-sided Fisher Exact Test P values < 0.05. Vertebrate: n = 146,555 variants in non-conserved target sites and n = 34,957 variants in conserved target sites. P = 4.3 x 10^{-15}. Mammalian: n = 183,560 variants in non-conserved target sites and n = 16,551 variants in conserved target sites. P = 1.6 x 10^{-5}. iMAPS scores for synonymous (green) and missense (orange) coding variants are shown on y-axis for reference.

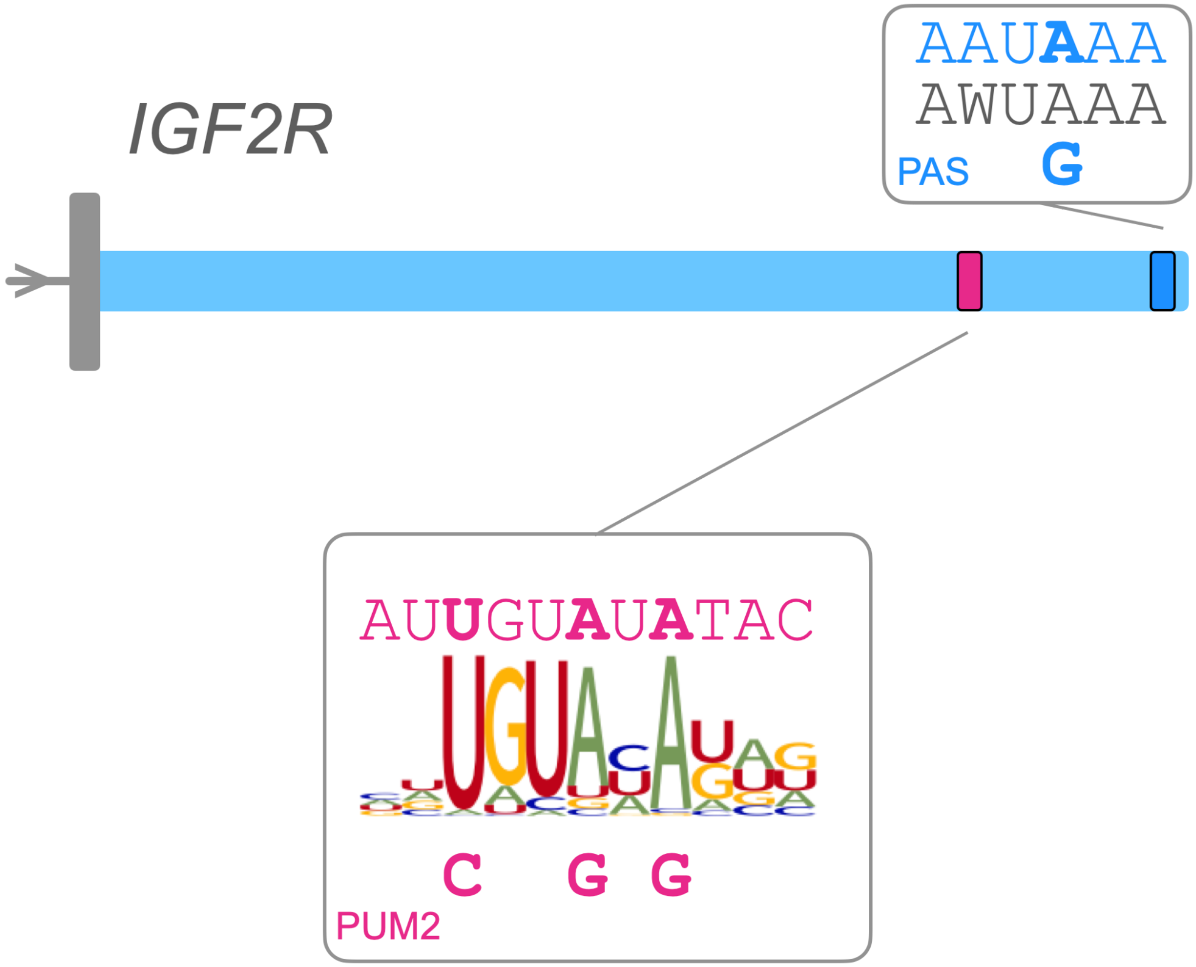

Supplementary Fig. 8: Highly disruptive variants in the *IGF2R* 3' UTR. Four 3' UTR gnomAD variants were labeled as disruptive*: three disrupting a single PUM2 ReP site, and one disrupting the PAS for the primary (and conserved) poly(A) site. For each element, the reference sequence is shown on the top with ancestral alleles in bold. Derived alleles are shown on the bottom. ReP sites are shown in pink, and PAS in blue. The top PAS hexamers are shown in gray. ReP site variants are shown in the context of an RBPamp affinity model for PUM2. *Note that the ReP site variants belong to a class with a minimum iMAPS of 0.05, while the PAS variant belongs to a class with a minimum iMAPS of 0.06.
