# Supplementary material for: Quantifying negative selection in human 3ʹ UTRs uncovers constrained targets of RNA-binding proteins
Source: Nat Commun. 2024 Jan 2;15:85. doi: 10.1038/s41467-023-44456-9 (PMC10762232; doi:10.1038/s41467-023-44456-9)
Supplement: Supplementary file 6 — Reporting Summary [file 41467_2023_44456_MOESM6_ESM.pdf]

nature portfolio

Corresponding author(s): Christopher B. Burge

Last updated by author(s): Dec 2 2023

Reporting Summary

Nature Portfolio wishes to improve the reproducibility of the work that we publish. This form provides structure for consistency and transparency in reporting. For further information on Nature Portfolio policies, see our Editorial Policies and the Editorial Policy Checklist.

Statistics

For all statistical analyses, confirm that the following items are present in the figure legend, table legend, main text, or Methods section.

n/a	Confirmed	
☐	☒	The exact sample size (*n*) for each experimental group/condition, given as a discrete number and unit of measurement
☒	☐	A statement on whether measurements were taken from distinct samples or whether the same sample was measured repeatedly
☐	☒	The statistical test(s) used AND whether they are one- or two-sided<br>*Only common tests should be described solely by name; describe more complex techniques in the Methods section.*
☒	☐	A description of all covariates tested
☐	☒	A description of any assumptions or corrections, such as tests of normality and adjustment for multiple comparisons
☐	☒	A full description of the statistical parameters including central tendency (e.g. means) or other basic estimates (e.g. regression coefficient) AND variation (e.g. standard deviation) or associated estimates of uncertainty (e.g. confidence intervals)
☐	☒	For null hypothesis testing, the test statistic (e.g. *F*, *t*, *r*) with confidence intervals, effect sizes, degrees of freedom and *P* value noted<br>*Give P values as exact values whenever suitable.*
☒	☐	For Bayesian analysis, information on the choice of priors and Markov chain Monte Carlo settings
☒	☐	For hierarchical and complex designs, identification of the appropriate level for tests and full reporting of outcomes
☐	☒	Estimates of effect sizes (e.g. Cohen's *d*, Pearson's *r*), indicating how they were calculated

Our web collection on statistics for biologists contains articles on many of the points above.

Software and code

Policy information about availability of computer code

Data collection	no software was used
Data analysis	R v4.2.0; Python v3.8.5; bedtools v2.29.1; vcftools v0.1.17; RBPamp (https://bitbucket.org/marjens/rbpamp); custom code (https://github.com/sfindlay11/iMAPS.git)

For manuscripts utilizing custom algorithms or software that are central to the research but not yet described in published literature, software must be made available to editors and reviewers. We strongly encourage code deposition in a community repository (e.g. GitHub). See the Nature Portfolio guidelines for submitting code & software for further information.

Data

Policy information about availability of data

All manuscripts must include a data availability statement. This statement should provide the following information, where applicable:

- Accession codes, unique identifiers, or web links for publicly available datasets
- A description of any restrictions on data availability
- For clinical datasets or third party data, please ensure that the statement adheres to our policy

Several publicly available datasets were utilized in this study and can be accessed as follows:
gnomAD variants; https://gnomad.broadinstitute.org/downloads
denovo db variants; https://denovo-db.gs.washington.edu/denovo-db/Download.jsp

Ancestral human genome sequence; Ensembl: https://ftp.ensembl.org/pub/current_fasta/ancestral_alleles/homo_sapiens_ancestor_GRCh38.tar.gz
DNase hypersensitivity peaks and CpG islands; UCSC Table Browser: https://genome.ucsc.edu/cgi-bin/hgTables
Gencode gene regions; UCSC Table Browser: https://genome.ucsc.edu/cgi-bin/hgTables, and Gencode: https://www.gencodegenes.org/human/release_32.html
H3K9me3 peaks; accessed using https://github.com/carjed/smaug-genetics/blob/master/download_ref_data.sh
CpG methylation data processed by the Roadmap Epigenomics Consortium can be requested from gnomAD
polA_DB data; https://exon.apps.wistar.org/PolyA_DB/v3/misc/download.php
RBPamp; https://bitbucket.org/marjens/rbpamp
ENCODE eCLIP and gene expression data; https://www.encodeproject.org/
Processed tamvar MPRA data; https://www.cell.com/cms/10.1016/j.cell.2021.08.025/attachment/d8a42d2a-9add-4815-b0ce-f4e1b28d5ca9/mmc1.xlsx
PhyloP scores; http://hgdownload.cse.ucsc.edu/goldenPath/hg38/phyloP100way/
eQTLs; https://www.gtexportal.org/home/
Annotated gene sets; https://github.com/QingboWang/gene_lists
ClinVar variants; UCSC Table Browser: https://genome.ucsc.edu/cgi-bin/hgTables
miRNA targets; https://www.targetscan.org/vert_80/vert_80_data_download/All_Target_Locations.hg19.bed.zip
Reporter assay RNA and plasmid read counts can be found in Supplementary Table 3.

Research involving human participants, their data, or biological material

Policy information about studies with human participants or human data. See also policy information about sex, gender (identity/presentation), and sexual orientation and race, ethnicity and racism.

Reporting on sex and gender	NA
Reporting on race, ethnicity, or other socially relevant groupings	NA
Population characteristics	NA
Recruitment	NA
Ethics oversight	NA

Note that full information on the approval of the study protocol must also be provided in the manuscript.

Field-specific reporting

Please select the one below that is the best fit for your research. If you are not sure, read the appropriate sections before making your selection.

☒ Life sciences ☐ Behavioural & social sciences ☐ Ecological, evolutionary & environmental sciences

For a reference copy of the document with all sections, see nature.com/documents/nr-reporting-summary-flat.pdf

Life sciences study design

All studies must disclose on these points even when the disclosure is negative.

Sample size	Sample sizes were based on available data
Data exclusions	Variants were filtered as described in the methods
Replication	Experimental results were reproduced across two independent replicates
Randomization	All reporter constructs were assayed in a single pool
Blinding	All reporter constructs were assayed in a single pool

Reporting for specific materials, systems and methods

We require information from authors about some types of materials, experimental systems and methods used in many studies. Here, indicate whether each material, system or method listed is relevant to your study. If you are not sure if a list item applies to your research, read the appropriate section before selecting a response.

Materials & experimental systems

n/a	Involved in the study	
☒	☐	Antibodies
☐	☒	Eukaryotic cell lines
☒	☐	Palaeontology and archaeology
☒	☐	Animals and other organisms
☒	☐	Clinical data
☒	☐	Dual use research of concern
☒	☐	Plants

Methods

n/a	Involved in the study	
☒	☐	ChIP-seq
☒	☐	Flow cytometry
☒	☐	MRI-based neuroimaging

Eukaryotic cell lines

Policy information about cell lines and Sex and Gender in Research

Cell line source(s)	HEK 293 cells provided by ATCC
Authentication	None of the cell lines used were authenticated
Mycoplasma contamination	All cell lines tested negative for mycoplasma
Commonly misidentified lines (See ICLAC register)	No commonly misidentified cell lines were used in this study
